# Control of Absence Seizures by the Thalamic Feed-Forward Inhibition

**DOI:** 10.3389/fncom.2017.00031

**Published:** 2017-04-26

**Authors:** Mingming Chen, Daqing Guo, Yang Xia, Dezhong Yao

**Affiliations:** ^1^Key Laboratory for NeuroInformation of Ministry of Education, School of Life Science and Technology, University of Electronic Science and Technology of ChinaChengdu, China; ^2^Center for Information in BioMedicine, University of Electronic Science and Technology of ChinaChengdu, China

**Keywords:** absence seizures, spike and wave discharges, corticothalamic network, feed-forward inhibition, mean-field model

## Abstract

As a subtype of idiopathic generalized epilepsies, absence epilepsy is believed to be caused by pathological interactions within the corticothalamic (CT) system. Using a biophysical mean-field model of the CT system, we demonstrate here that the feed-forward inhibition (FFI) in thalamus, i.e., the pathway from the cerebral cortex (Ctx) to the thalamic reticular nucleus (TRN) and then to the specific relay nuclei (SRN) of thalamus that are also directly driven by the Ctx, may participate in controlling absence seizures. In particular, we show that increasing the excitatory Ctx-TRN coupling strength can significantly suppress typical electrical activities during absence seizures. Further, investigation demonstrates that the GABA_A_- and GABA_B_-mediated inhibitions in the TRN-SRN pathway perform combination roles in the regulation of absence seizures. Overall, these results may provide an insightful mechanistic understanding of how the thalamic FFI serves as an intrinsic regulator contributing to the control of absence seizures.

## Introduction

Absence epilepsy is a common subtype of idiopathic generalized epilepsies and mainly occurs in the childhood years (Durón et al., 2005; Tolaymat et al., 2015). This chronic neurological brain disorder is characterized by recurrent absence seizures, often leading to sudden periods of impaired consciousness (Crunelli and Leresche, 2002). During its ictal period, the bilaterally synchronous 2–4 Hz spike and wave discharges (SWDs) can be observed on the electroencephalogram (EEG) of patients, which are regarded as the electrophysiological hallmark of absence seizures (Crunelli and Leresche, 2002). Despite that onset mechanisms of absence seizures still remain debated, accumulating evidence indicates that the emergence of 2–4 Hz SWDs during absence seizures critically depends on cerebral cortex (Ctx) and thalamus (Blumenfeld, 2005; Bazhenov et al., 2008; Lytton, 2008; Avoli, 2012; Holt and Netoff, 2013; Depaulis et al., 2016). It has been postulated that properly disrupting abnormal interactions within corticothalamic (CT) system can cause SWD suppression, thus preventing absence seizures.

Previous studies have shown that direct neuronal modulations within the CT system may destabilize the pathological oscillations during absence seizures (Suffczynski et al., 2004; Lüttjohann and van Luijtelaar, 2013; Paz et al., 2013; Taylor et al., 2014, 2015; Fan et al., 2015; Liu et al., 2016). The most frequently reported direct stimulation site is the thalamus. Past experimental evidence has suggested that both closed-loop optogenetic control of thalamus and high frequency thalamic stimulation can dramatically inhibit the occurrence of epileptic seizures (Lüttjohann and van Luijtelaar, 2013; Paz et al., 2013). In addition to these direct interventions, indirect neuronal modulations from several external brain regions have also been identified to be effective in controlling absence seizures (Deransart et al., 1998; Paz et al., 2007; Chen et al., 2014, 2015; Kros et al., 2015a,b). As an intermediate bridge between the Ctx and thalamus, the basal ganglia (BG) are widely considered as an ideal modulation site to prevent absence seizures (Depaulis et al., 1994; Deransart et al., 1998; Paz et al., 2007; Luo et al., 2012). This hypothesis is supported by both experimental and computational evidence, showing that the BG might multiply control absence seizures through the GABAergic nigro-thalamic and pallido-cortical pathways (Deransart et al., 1998; Paz et al., 2005, 2007; Luo et al., 2012; Chen et al., 2014, 2015; Hu et al., 2015; Hu and Wang, 2015). Besides the BG, another ideal candidate to modulate abnormal CT oscillations is the cerebellar nuclei (CN; Kros et al., 2015a,b). Using a mouse model of absence epilepsy, a recent study has found that increasing the frequency and regularity of CN neuronal firing can significantly reduce the occurrence of SWDs and terminate absence seizures (Kros et al., 2015b).

Moreover, recent intensive statistical investigations on neuronal connectivity have revealed that the brain contains some recurring nontrivial patterns of interconnected neurons, termed as “microcircuit motifs” (Sporns and Kötter, 2004; Womelsdorf et al., 2014; Paz and Huguenard, 2015). Interestingly, these microcircuit motifs are found to be linked to each other in a way that does not spoil the independent function of each microcircuit motif, thus postulating to work as basic building blocks of nervous systems and perform critical functional roles in regulating neurodynamics at the circuit level (Sporns and Kötter, 2004; Li, 2008; Guo and Li, 2009; Potjans and Diesmann, 2014; Womelsdorf et al., 2014; Paz and Huguenard, 2015; Yuste, 2015). As one of the most significant microcircuit motifs, the feed-forward inhibition (FFI) has been observed in multiple brain regions, including the neocortex, thalamus, hippocampal, and basal ganglia (Sloviter, 1991; Sun et al., 2005; Assisi et al., 2007; Tepper et al., 2008; Kee et al., 2015; Paz and Huguenard, 2015; Khubieh et al., 2016; Lindahl and Hellgren Kotaleski, 2016). Emerging evidence has suggested that significant loss of FFI in the brain can cause an imbalance between excitation and inhibition of neurons toward excessive excitation (Paz and Huguenard, 2015). Such imbalanced excitation-inhibition might lead to hypersynchronous neural firing activities that are commonly involved in various types of epileptic seizures.

Neurons in the thalamic reticular nucleus (TRN) receive excitatory signals from the Ctx, and send both GABA_A_- and GABA_B_-mediated inhibitory signals to the specific relay nuclei (SRN) of thalamus, which are also driven by the excitatory signals from the Ctx. Anatomically, these neural projections together form the so-called thalamic FFI microcircuit motif (Pinault, 2004; Paz and Huguenard, 2015). Although past experimental studies have suggested that restoring cortical excitation in TRN neurons may prevent absence seizures (Paz et al., 2011; Paz and Huguenard, 2015), so far the detailed biophysical mechanisms of SWD suppression induced by this thalamic FFI is still poorly understood. In the present study, we utilize a mean-field CT network model to address this question. We find that both the excitatory Ctx-TRN coupling strength and the relative strength between the GABA_B_- and GABA_A_-mediated inhibitions in the TRN-SRN pathway play critical roles in preventing pathological 2–4 Hz SWDs generated by the CT system. These findings highlight the functional importance of thalamic FFI in controlling absence seizures, and might provide insights into the treatment of absence epilepsy.

## Methods and analysis

### Computational model of the CT network

**Figure 1:**
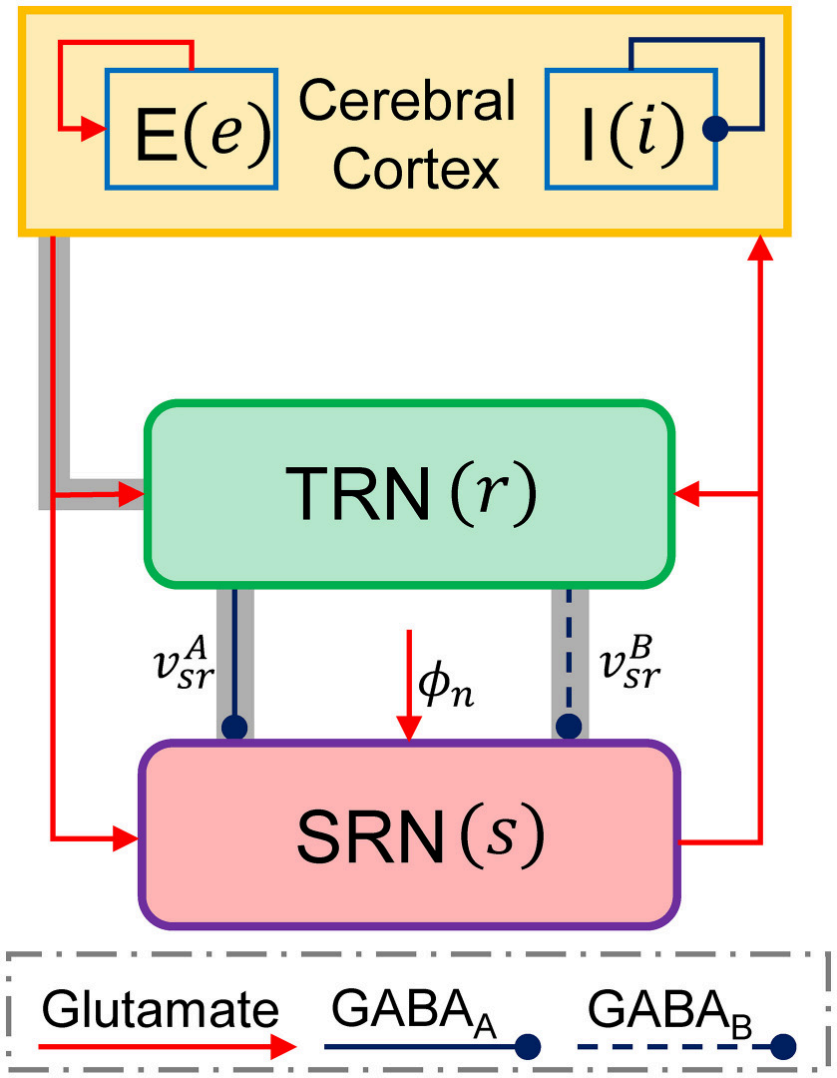
**The schematic framework of the corticothalamic network**. There are four neural populations that the excitatory pyramidal neural population (*e*), the inhibitory interneuron population (*i*), the TRN (*r*), and SRN (*s*) in the corticothalamic network. A non-specific external input to the SRN is denoted as ϕ_*n*_. The connections within the network are mainly mediated by excitatory glutamate described by red lines with arrow heads and inhibitory GABA denoted by blue lines with round heads. The solid and dashed lines from the TRN to SRN are used to distinguish the projections mediated by GABA_A_ ($v_{sr}^{A}$) and GABA_B_ ($v_{sr}^{B}$) synapses, respectively. The gray shaded pathways show the thalamic feed-forward inhibition (FFI).

Previous studies have suggested that the generation of 2–4 Hz SWDs during absence seizures may be caused by abnormal interactions between the Ctx and thalamus (Crunelli and Leresche, 2002; Robinson et al., 2002; Suffczynski et al., 2004; Blumenfeld, 2005; Breakspear et al., 2006; Lytton, 2008; Marten et al., 2009a). To investigate possible roles of the thalamic FFI in controlling absence seizures, we establish a macroscopic-level computational model that describes the population dynamics of the CT network. As shown in Figure 1, our CT network comprises four neural populations, which are indicated as follows: *e* = excitatory pyramidal neurons, *i* = inhibitory interneurons, *r* = TRN and *s* = SRN. Inspired by anatomical data, three types of neural projections are considered in our CT model (Figure 1). The blue solid and dashed lines with round heads are used to represent the inhibitory projections mediated by GABA_A_ and GABA_B_ synapses, respectively. The red lines with arrow heads are employed to denote the glutamate-mediated excitatory projections. Similar to previous modeling studies, a non-specific external input ϕ_*n*_ is also injected to the SRN to mimic the sensory input (Robinson et al., 2002; Breakspear et al., 2006; Marten et al., 2009a; Chen et al., 2014, 2015). Obviously, our CT network contains the thalamic FFI that lies at the Ctx-TRN-SRN pathway (see the gray shaded pathways in Figure 1).

In the present study, each neural population in the CT network is simulated using the mean-field model proposed by Robinson and his colleagues (Robinson et al., 2002; Breakspear et al., 2006). For a given neural population *a*, the population dynamics can be characterized by three state variables: the mean membrane potential *V*_*a*_, the mean firing rate *Q*_*a*_, and the presynaptic activity ϕ_*a*_. The transformation from the mean membrane potential *V*_*a*_ to the mean firing rate *Q*_*a*_ obeys a sigmoid function (Robinson et al., 2002; Breakspear et al., 2006; Marten et al., 2009a; Freyer et al., 2011; Yang et al., 2016):

(1) $$Q_{a}(\text{r},t)\equiv F[V_{a}(\text{r},t)]=\frac{Q_{a}^{\max}}{1+\exp\left[-\frac{\pi }{\sqrt{3}}\frac{\left(V_{a}(\text{r},t)-\theta_{a}\right)}{\sigma }\right]},$$

where subscripts *a* ∈ *A* = {*e, i, r, s*} indicate the neural populations considered in the CT network. r represents the spatial position in the brain, θ_*a*_ is the threshold of the mean firing, and σ denotes the standard deviation of the firing rate. Due to the boundedness of the sigmoid function, the maximum firing rate $Q_{a}^{\max}$ in the Equation (1) ensures the mean firing rate *Q*_*a*_ within the physiological range as changing the mean membrane potential *V*_*a*_.

Theoretically, the filtered incoming postsynaptic potentials from presynaptic neural populations in the dendrites lead to fluctuations of the mean membrane potential *V*_*a*_ at the position r. This dynamical process can be modeled as (Robinson et al., 2002; Breakspear et al., 2006; Marten et al., 2009a; Freyer et al., 2011):

(2) $$D_{\alpha \beta }V_{a}(\text{r},t)=\sum_{b\in A}v_{ab}\cdot \phi_{b}(\text{r},t),$$

(3) $$D_{\alpha \beta }=\frac{1}{\alpha \beta }[\frac{\partial^{2}}{\partial t^{2}}+(\alpha +\beta )\frac{\partial }{\partial t}+\alpha \beta ].$$

Here, the *D*_αβ_ is a differential operator describing the synaptic and dendritic filtering of incoming signals. The inverses of α and β denote the decay and rise time constants of membrane potential caused by incoming signals, respectively. *v*_*ab*_ describes the coupling strength from the neural population *b* to *a*, and ϕ_*b*_(r, *t*) represents the presynaptic activity of the neural population *b*. Similar to previous modeling studies (Chen et al., 2014, 2015), the transmission delays among most neural populations are ignored in our model. Note that, however, we introduce a delay parameter τ to the GABA_B_-related incoming pulse rate [i.e., ϕ_*r*_(r, *t* − τ)] to mimic the slow kinetics of GABA_B_ receptor-mediated inhibition.

In the Ctx, the propagation effects of excitatory firing rates *Q*_*e*_ along the cortical surface satisfy the damped wave equation (Robinson et al., 2002; Breakspear et al., 2006; Marten et al., 2009a; van Albada and Robinson, 2009; van Albada et al., 2009; Freyer et al., 2011; Chen et al., 2014, 2015):

(4) $$\frac{1}{\gamma_{e}^{2}}[\frac{\partial^{2}}{\partial t^{2}}+2\gamma_{e}\frac{\partial }{\partial t}+\gamma_{e}^{2}-v_{e}^{2}\nabla^{2}]\phi_{e}(\text{r},t)=Q_{e}(\text{r},t),$$

where ∇^2^ represents the Laplacian operator (the second spatial derivative) and ϕ_*e*_ denotes the electrical field arising from the cortical excitatory neurons firing propagating along axons. The temporal damping rate of pulses is determined by the γ_*e*_ = *v*_*e*_/*r*_*e*_, where *v*_*e*_ is the conduction velocity and *r*_*e*_ denotes the characteristic range of axons of excitatory neurons in the Ctx. For other neural populations, their axons are too short to support wave propagation on the relevant scales. This indicates ϕ_*c*_ = *F*(*V*_*c*_) (*c* = *i, r, s*). On the other hand, because absence seizures are believed to occur simultaneously throughout the brain, it is reasonable to assume that the spatial activities during absence seizures are uniform in our CT model. We therefore ignore the spatial derivative in Equation (4), and the propagation effect of cortical excitatory axonal field can be rewritten as (Breakspear et al., 2006; Marten et al., 2009a; Freyer et al., 2011):

(5) $$\frac{1}{\gamma_{e}^{2}}[\frac{\partial^{2}}{\partial t^{2}}+2\gamma_{e}\frac{\partial }{\partial t}+\gamma_{e}^{2}]\phi_{e}(t)=Q_{e}(t).$$

We can further simplify our model by assuming that the mean membrane potential *V*_*i*_ and mean firing rate *Q*_*i*_ of the inhibitory interneuron population satisfy *V*_*i*_ = *V*_*e*_ and *Q*_*i*_ = *Q*_*e*_. Note that this assumption is reasonable, because the intracortical connections are suggested to be proportional to the number of synapses involved (Robinson et al., 2002, 2004; Breakspear et al., 2006; Marten et al., 2009a; van Albada and Robinson, 2009; van Albada et al., 2009; Freyer et al., 2011; Chen et al., 2014, 2015; Hu et al., 2015). Consequently, we can rewrite our mean-field CT model in the first-order form, as given in the Appendix. It should be noted that the model parameters in the established CT network are adapted from previous studies (Robinson et al., 2002, 2004; Breakspear et al., 2006; Marten et al., 2009a; van Albada and Robinson, 2009; van Albada et al., 2009; Freyer et al., 2011; Chen et al., 2014, 2015; Hu et al., 2015), which are systematically summarized in Table 1. These default parameters are estimated from real physiological data. Unless stated otherwise, we use these default parameter values in the following studies.

**Table 1:** **Model default parameters**.

**Symbol**	**Value**	**Unit**	**Description**
$Q_{e}^{\max},Q_{i}^{\max}$	250	Hz	Cortical maximum firing rate
$Q_{s}^{\max}$	250	Hz	SRN maximum firing rate
$Q_{r}^{\max}$	250	Hz	TRN maximum firing rate
θ_*e*_, θ_*i*_	15	mV	Mean firing threshold of cortical populations
θ_*s*_	15	mV	Mean firing threshold of SRN
θ_*r*_	15	mV	Mean firing threshold of TRN
*v*_*ee*_	1	mV s	Coupling strength from PY to PY
−*v*_*ei*_	1.8	mV s	Coupling strength from IIN to PY
*v*_*re*_	0.05	mV s	Coupling strength from PY to TRN
*v*_*rs*_	0.5	mV s	Coupling strength from SRN to TRN
$-v_{sr}^{A,B}$	0.8	mV s	Coupling strength from TRN to SRN
*v*_*es*_	1.8	mV s	Coupling strength from SRN to PY
*v*_*se*_	2.4	mV s	Coupling strength from PY to SRN
γ_*e*_	100	Hz	Cortical damping rate
τ	50	ms	Time delay due to slow synaptic kinetics of GABA_B_
α	50	s^−1^	Synaptodendritic decay time constant
β	200	s^−1^	Synaptodendritic rise time constant
σ	6	mV	Threshold variability of firing rate
*v*_*sn*_ϕ_*n*_	2	mV	Non-specific subthalamic input onto SRN

*Model parameters employed in the present study are adapted from previous studies (Chen et al., 2014, 2015) and references cited therein. Unless otherwise noted, we use these default parameter values for simulations. Note that PY and IIN denote excitatory pyramidal neurons and inhibitory interneurons in the cerebral cortex, respectively*.

In simulations, our computational model is implemented with custom codes written in Matlab (MathWorks). We use the standard fourth-order Runge-Kutta method for numerically integrating normal differential equations, and employ an algorithm adapted from Matlab dde23 for calculating the delay differential equations (Shampine and Thompson, 2001). The temporal resolution of integration is fixed at 0.05 ms. Additional simulations confirm that this time step is sufficiently small to ensure an accurate simulation of our established CT model. All simulations are carried out for sufficiently long time to collect stable data for further analysis. The computer codes implementing our CT model will be available upon request via email.

### Data analysis

**Figure 2:**
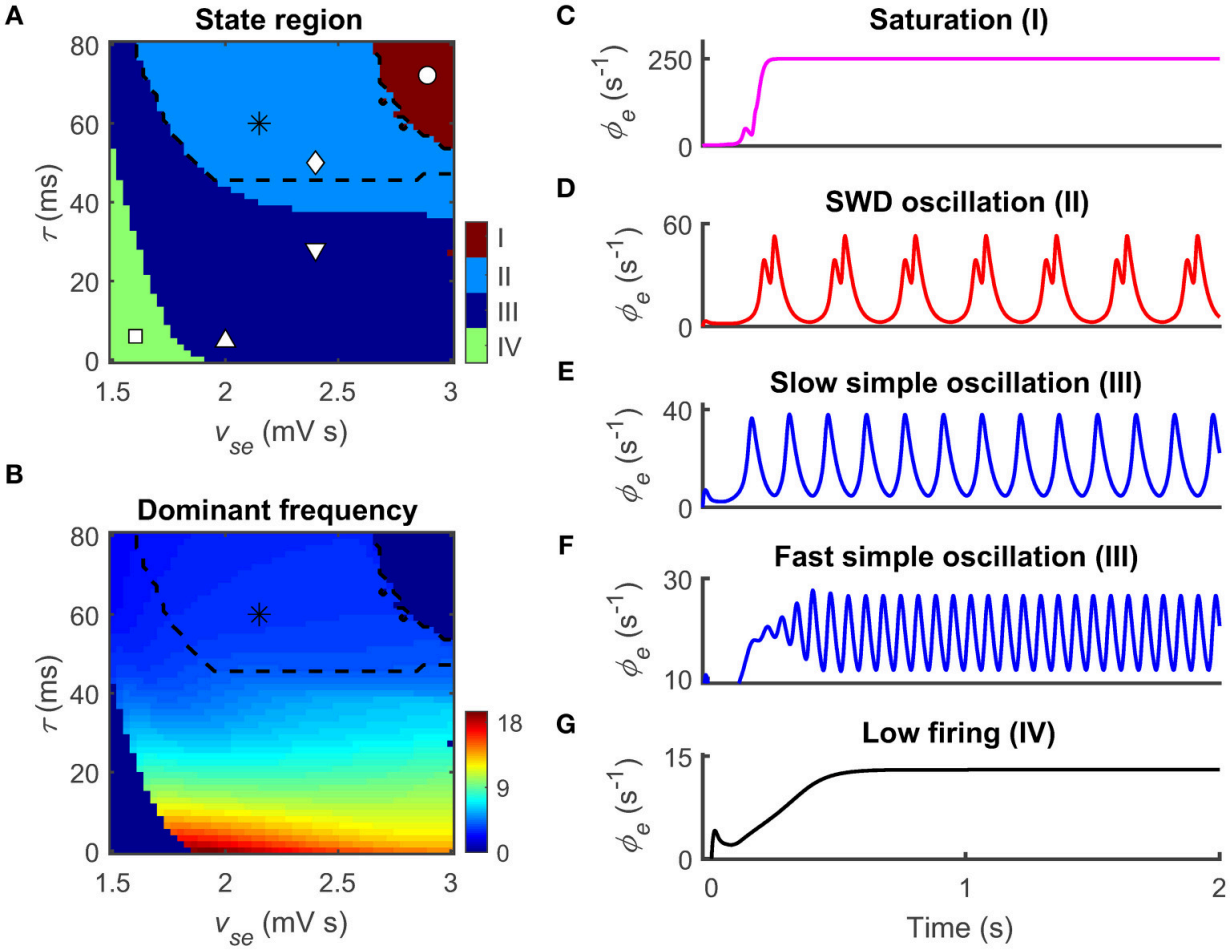
**Two epileptogenic factors inducing absence seizures in the corticothalamic network**. The coupling strength *v*_*se*_ that from the cerebral cortex to the SRN and the time delay τ caused by the slow kinetics of GABA_B_ receptors are two epileptogenic factors for inducing absence seizures. **(A,B)** Distributions of dynamical states **(A)** and dominant frequency **(B)** in the two-parameter space (*v*_*se*_, τ). Four dynamical states could be observed: the saturation (I), the SWD oscillation (II), the simple oscillation (III) that includes slow and fast simple oscillations, and the low firing (IV). The regions of the typical 2–4 Hz SWDs indicated with asterisk (“^*^”) are surrounded by black dashed lines both in **(A)** and **(B)**. **(C–G)** Five time series of ϕ_*e*_ are used to show the different firing patterns and the corresponding values are given by I (“ ◦ ”), II (“ ◊ ”), III (“ ∇ ” and “ Δ ”), and IV (“ □ ”) in **(A)**.

**Figure 3:**
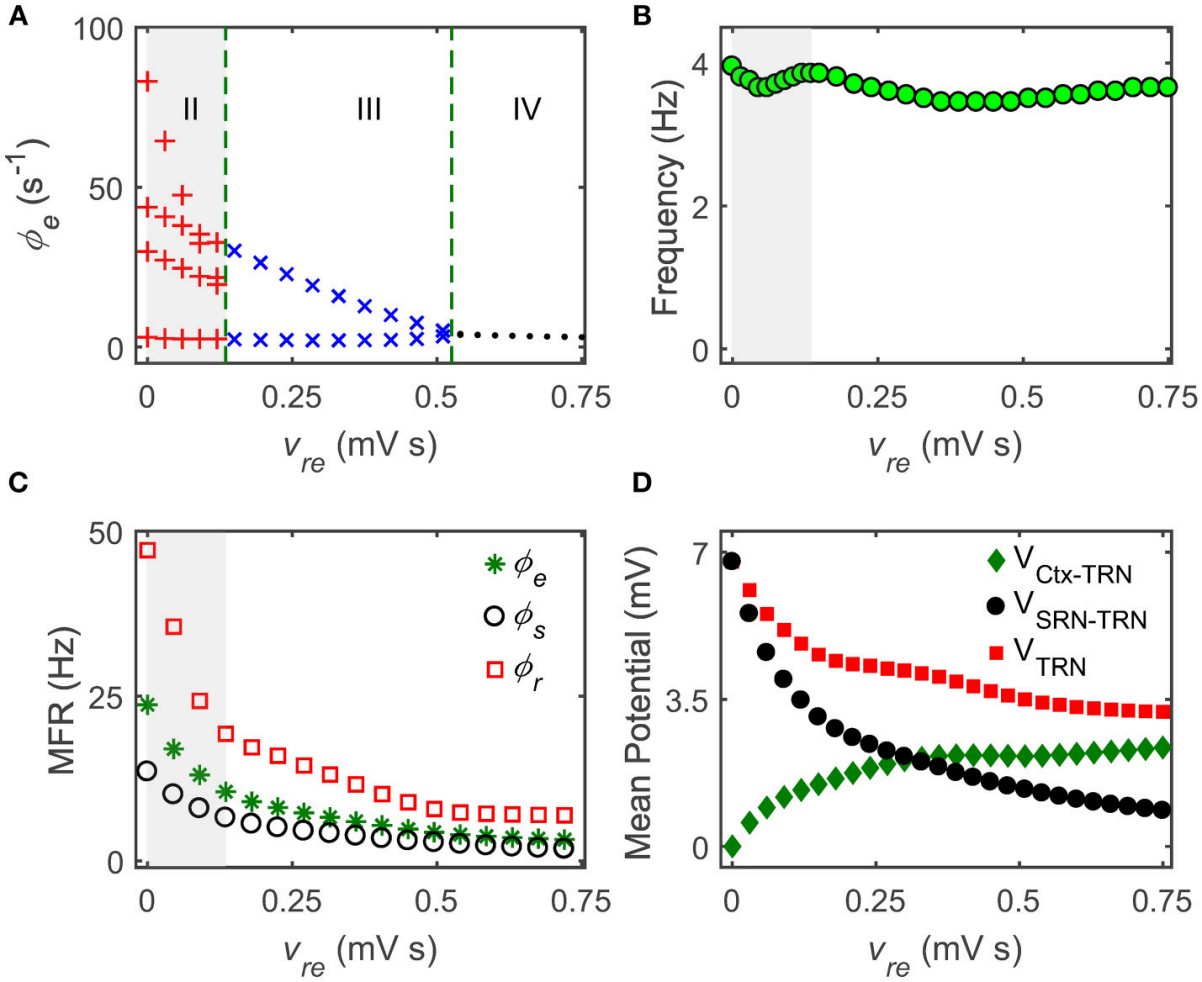
**The Ctx-TRN pathway contributes to controlling absence seizures. (A)** State analysis of ϕ_*e*_ as a function of the coupling strength of the Ctx-TRN pathway *v*_*re*_. Enhancing the coupling strength *v*_*re*_ could push the firing pattern from the SWD oscillation (II) to the simple oscillation (III) and to the low firing (IV). **(B)** The dominant frequency of the cortical oscillations ϕ_*e*_ changes with the enhancement of *v*_*re*_. **(C)** The mean firing rates (MFRs) of three neural populations that the Ctx (“^*^”), the SRN (“ ◦ ”), and TRN (“ □ ”) as a function of *v*_*re*_. The gray regions in **(A–C)** describe the regions of the typical 2–4 Hz SWDs, and the dashed green lines are used to show the demarcations of different dynamical states in the control process of absence seizures. **(D)** Changes of mean potential as a function of *v*_*re*_. *V*_Ctx-TRN_ denotes the mean potential from the Ctx to TRN, *V*_SRN-TRN_ represents the mean potential from the SRN to TRN, *V*_TRN_ shows the mean potential of TRN.

In the present study, we analyze the simulated data generated by our model from various aspects. Note that these data analysis techniques have been described in our previous studies (Chen et al., 2014, 2015). To classify the dynamical states of cortical oscillations, we determine the stable local maximum and minimum values of cortical excitatory axonal fields occurring in one cortical oscillatory period for each experimental setting. For a specific parameter, the state analysis of cortical oscillations is plotted as a function of this critical parameter (for example, see Figure 3). By employing this state analysis technique, we can identify the regions of different dynamical states in the two-dimensional parameter space (for example, see Figure 2). To evaluate the oscillation frequency of cortical oscillations, we compute the power spectral density of the time series ϕ_*e*_ using the Fast Fourier transform. For an experimental setting, the maximum peak frequency emerging in the power spectral density is defined as the dominant frequency of cortical oscillations. By combining both the state and frequency analysis techniques, the boundaries of the typical 2–4 Hz SWDs regions can be further outlined in the two-dimensional parameter space (see the asterisk regions in Figure 2). To understand the biophysical mechanisms underlying the control of absence seizures by the thalamic FFI, we calculate the long-term mean firing rates (MFRs) of three populations that the Ctx, the TRN, and SRN. To quantify impacts of Ctx and SRN on TRN, we further compute the mean potential that from Ctx and SRN to TRN, denoted as *V*_Ctx-TRN_ and *V*_SRN-TRN_ in Figure 3D. In this work, *V*_Ctx-TRN_ is defined as the product of the *v*_*re*_ and the MFRs of Ctx, whereas *V*_SRN-TRN_ represents the product of the *v*_*rs*_ and the MFRs of SRN. The summation of *V*_Ctx-TRN_ and *V*_SRN-TRN_ is thus defined as the overall effect of Ctx and SRN on TRN (*V*_TRN_).

## Results

### Our CT model can generate typical 2–4 Hz SWDs by introducing pathological mechanisms

Accumulating experimental data reveal that the generation of absence seizures is tightly associated with abnormal interactions within the CT network (Snead, 1995; Danober et al., 1998). Specifically, both the strong excitatory projections from the Ctx to the SRN and the slow kinetics of GABA_B_ synapses along the TRN-SRN pathway are believed to be critical pathological factors in absence seizures (Destexhe, 1998; Robinson et al., 2002; Breakspear et al., 2006; Marten et al., 2009a,b; Han et al., 2012). Theoretically, the strong coupling strength of the cortico-thalamic pathway tends to induce the high amplitude oscillations, and the slow kinetics of GABA_B_ synapses act as a delayed low-pass filter and can be linked to the production of slow waves. Thus, simultaneous modulation of these two parameters may destroy the normal oscillatory pattern generated in the CT system, triggering the onset of absence seizures.

To examine whether these pathological factors are also available in our CT model, we employ two related parameters: the excitatory coupling strength *v*_*se*_ and the GABA_B_ delay τ. Figures 2A,B show the state and frequency analysis in the combined parameter space (*v*_*se*_, τ), respectively. Consistent with previous findings (Chen et al., 2014, 2015), the model exhibits four types of dynamical states with different frequency characteristics. The first dynamical state is the saturation firing that occurs for sufficiently long τ and strong *v*_*se*_ (region I in Figure 2A). Under this condition, inhibitions from the TRN to SRN cannot successfully suppress the excessive excitation caused by strong cortico-thalamic interactions. Such excessive excitation drives the firing of cortical neurons to increase from the low firing to the maximum firing in a short time (Figure 2C). For a long GABA_B_ delay τ, the SWD oscillation state appears provided that the excitatory coupling strength *v*_*se*_ is appropriately strong. In this case, the TRN-SRN inhibitions mediated by GABA_A_ and GABA_B_ receptors effectively shape SRN firing at different instants. This dual suppression creates double firing peaks for SRN neurons, further leading to the generation of cortical SWDs (region II in Figure 2A). Importantly, most of SWD oscillation region is contained in the typical 2–4 Hz frequency range (asterisk regions in Figures 2A,B), which can be comparable with EEG signals of real patients during absence seizures. On the other hand, the decrease in delay τ mixes the GABA_A_- and GABA_B_-induced inhibitions, weakening their double peak shaping effect. For a fixed *v*_*se*_, we therefore observe that the network dynamics can transit from the SWD oscillation state to the simple oscillation state by lowing the GABA_B_ delay (region III in Figure 2A). Theoretically, the boundary between dual time scales and mixed time scale highly depends on the decay and rise time constants of membrane potential in our model (Chen et al., 2014). For each GABA_A_-induced inhibition caused by TRN neurons, the SRN neurons require a certain level of recovery time to restore their neuronal firing to the rising state. If this recovery time is shorter than the GABA_B_ delay, another firing peak can be created to SRN neurons due to the latter GABA_B_-induced inhibition. Further, frequency analysis reveals that the dominant frequency of simple oscillation state relies on the GABA_B_ delay. The shorter the delay parameter τ, the faster the simple oscillations generated by our model (Figures 2E,F). However, if the excitatory coupling strength *v*_*se*_ is too weak, neural oscillations within the CT network cannot be maintained. In this case, the network dynamics is kicked into the low firing state (region IV in Figures 2A,G).

These findings demonstrate that our model can replicate different dynamical states of human brain. Remarkably, the model successfully generates the typical 2–4 Hz SWDs by introducing previous pathological mechanisms. In the following studies, we set *v*_*se*_ = 2.4 mV s and τ = 50 ms as default, and explore the effects of the thalamic FFI on controlling absence seizures.

### Roles of the Ctx-TRN pathway in controlling absence seizures

In the brain, the FFI microcircuits are believed to serve as fundamental regulators in balancing neural excitation and inhibition. It is widely accepted that dysfunction of FFI microcircuits might lead to excessive and/or hypersynchronous neural activities and evoke epileptic seizures (Paz and Huguenard, 2015). Importantly, a recent experimental study also showed a specific reduction in Ctx-TRN strength in the GluA4-deficient (Gria4^−/−^) mice model of absence epilepsy (Paz et al., 2011). We thus hypothesize that the classical thalamic FFI microcircuit, i.e., the Ctx-TRN-SRN pathway (Figure 1, gray lines), may participate in the modulation of absence seizures.

To theoretically validate whether this hypothesis is correct, we concentrate on the TRN and modulate its activation level by changing the coupling strength of the excitatory Ctx-TRN pathway. Figure 3A shows the one-dimensional state analysis as a function of the Ctx-TRN strength *v*_*re*_. Indeed, we find that the Ctx-TRN strength performs an active role in terminating absence seizures. A slight enhancement of the Ctx-TRN strength from its default value can suppress the SWD oscillations and push the model dynamics into the simple oscillation state (Figure 3A). For sufficiently strong strength *v*_*re*_, the model dynamics is even kicked into the low firing state (Figure 3A), implying a strong suppression effect due to the strengthened Ctx-TRN pathway. To characterize the frequency feature of model dynamics, the dominant frequency of neural oscillations is estimated by using the spectral analysis technique. For equivalent GABA_A_- and GABA_B_-mediated inhibitions in the TRN-SRN pathway, we observe that the dominant frequency of neural oscillations is stable and insensitive to the excitatory Ctx-TRN strength (Figure 3B).

To understand in detail how the Ctx-TRN pathway inducing SWD suppression arises, we further plot the MFR vs. the excitatory Ctx-TRN pathway strength *v*_*re*_ for key neural populations in Figure 3C. As expected, the enhancement of excitatory coupling strength *v*_*re*_ tends to excite TRN neurons (Figure 3D, green, indicted by diamonds). However, increasing the TRN activation suppresses the firing of SRN, which subsequently inhibits cortical neurons. Conversely, however, inactivation of SRN neurons will introduce a collision to TRN neurons and reduce their firing in a significant way (Figure 3D, black and red, indicated by circles and squares, respectively). By strengthening the Ctx-TRN pathway, this collision destabilizes the pathological balance and causes an overall inhibition effect in the CT system (Figure 3C). Such inhibition effect on TRN weakens the double peak shaping effect due to the slow kinetics of GABA_B_ receptors. For a relatively strong excitatory Ctx-TRN strength, this GABA_B_ weakening effect is considerable and therefore the generation of SWDs is terminated in our model.

**Figure 4:**
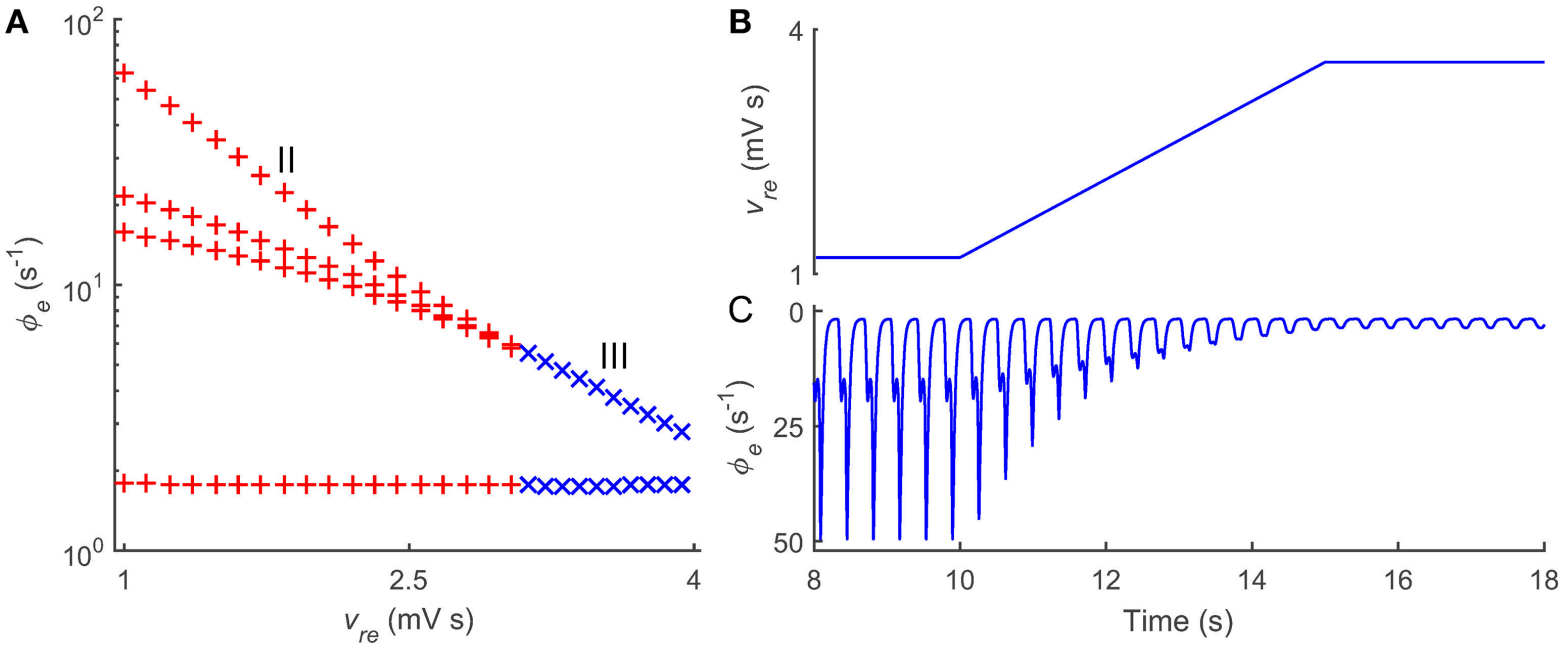
**Roles of thalamic FFI in controlling absence seizures induced by transmission delays within corticothalamic loop. (A)** State analysis of ϕ_*e*_ as a function of the coupling strength *v*_*re*_. The enhancement of the coupling strength *v*_*re*_ could push the firing pattern from the SWD oscillation (II) to the simple oscillation (III). **(B)** Linearly enhancing the Ctx-TRN pathway (*v*_*re*_) to observe the dynamical control process of absence seizures. **(C)** The changes of cortical oscillations ϕ_*e*_ with linearly increasing *v*_*re*_. Note that in these simulations, the modified parameters are τ_0_/2 = 40 ms, *v*_*es*_ = 3.2 mV s, *v*_*se*_ = 3.4 mV s, *v*_*re*_ = 1.2 ~ 3.6 mV s, and *v*_*sn*_ϕ_*n*_ = 8.0 mV.

Past studies have demonstrated that several other pathological factors may also lead to typical 2–4 Hz SWD oscillations in the CT system (Robinson et al., 2002; Breakspear et al., 2006). Thus, a natural question is whether such control feature caused by the thalamic FFI is a generalized regulatory mechanism for absence seizures. We try to answer this question by introducing another SWD generation mechanism into our model. In literature, it has been widely reported that long transmission delay between the Ctx and thalamus can drive the CT system to produce the 2–4 Hz SWDs. To apply this pathological factor in our model, we block the GABA_B_ pathway from TRN to SRN, and consider a bidirectional transmission delay τ_0_/2 between the Ctx and thalamus. Note that several coupling strengths are also adapted to ensure the CT system to generate the SWD oscillation. Our results presented in Figure 4 confirm that the increase in the excitatory coupling strength *v*_*re*_ can successfully suppress the typical 2–4 Hz SWDs due to abnormal transmission delay between the Ctx and thalamus, further emphasizing the generality and functional importance of the thalamic FFI in regulating absence seizures.

Taken together, we show that the thalamic FFI indeed participates in the modulation of absence seizures. Our theoretical observations indicate that normal CT oscillations require a certain level of Ctx-TRN strength, and loss of the Ctx-TRN strength might lead to over-excitation in the CT system and thus cause absence seizures. Importantly, this control manner by the thalamic FFI is found to be a possibly generalized regulatory mechanism for absence seizures and may be extendable to other pathological factors.

### Combination roles of GABA_A_- and GABA_B_-mediated inhibitions in the TRN-SRN pathway in controlling absence seizures

The TRN-SRN pathway is another component of the thalamic FFI and might therefore also play essential roles in controlling absence seizures. Because neural projections from the TRN to the SRN are mediated by both GABA_A_ and GABA_B_ receptors, we next investigate effects of these two types of inhibitions on the modulation of absence seizures.

**Figure 5:**
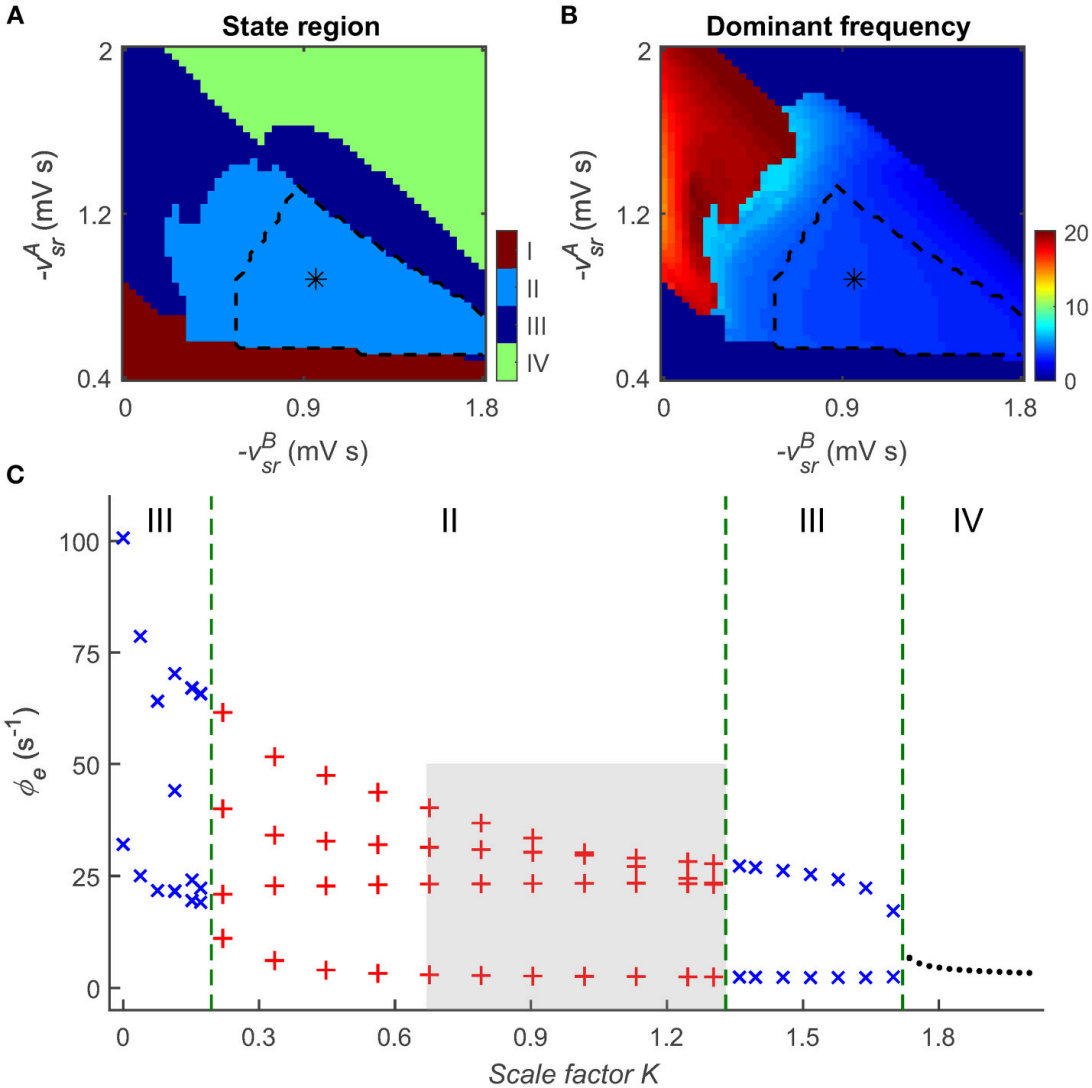
**Combination roles of GABA_**A**_- and GABA_**B**_-mediated inhibitions in the TRN-SRN pathway in controlling absence seizures**. The distributions of dynamical states **(A)** and dominant frequency **(B)** in the two-parameter space ($-v_{sr}^{B},-v_{sr}^{A}$). Similar to the results in Figure 2A, there are four dynamical states in this two-parameter space, the regions of the typical 2–4 Hz SWDs indicated with asterisk (“*”) are surrounded by black dashed lines. **(C)** State analysis of ϕ_*e*_ as a function of the scale factor *K*, defined as the relative strength of GABA_B_ and GABA_A_ inhibitions in the TRN-SRN pathway. The gray region describes the region of typical 2–4 Hz SWDs, and the dashed green lines are used to show the demarcations of different dynamical states in the control process of absence seizures.

Figure 5A depicts the two-dimensional state analysis in the ($-v_{sr}^{B},-v_{sr}^{A}$) panel. Similarly, we identify that four types of dynamical states are distributed in these two-parameter space (Figure 5A), revealing that the dynamics of the CT system depends on both GABA_A_ and GABA_B_ inhibitions. At a low level of GABA_A_ inhibition, the CT system exhibits the saturation firing state (Figure 5A, region I). For a relatively larger GABA_B_ delay that we considered in this work (Destexhe, 1998; Marten et al., 2009a,b), this occurs even when the GABA_B_ inhibition is at a sufficiently high level, indicating that strong disinhibition of GABA_A_ receptors in the TRN-SRN pathway may cause explosion of high-rate activities (Chen et al., 2014). Note that, however, such effect caused by GABA_A_ inhibition might be also dependent on the delay time in the GABA_B_ inhibition. If both GABA_A_ and GABA_B_ inhibitions are too strong, the firing of SRN neurons is extremely inhibited. In this case, the network dynamics is pushed into the low firing state (Figure 5A, region IV). As a consequence, neural oscillations of the CT system can be observed for intermediate GABA_A_ and GABA_B_ inhibitions (Figure 5A, regions II and III). Moreover, we find that the emergence of SWDs is highly associated with a scale factor *K*, defined as the relative strength of GABA_B_ and GABA_A_ inhibitions in the TRN-SRN pathway. For a fixed GABA_A_ strength $-v_{sr}^{A}$, an intermediate *K* might cause SWDs in the CT system (Figure 5C), thus might serve as a possible pathological factor of absence seizures.

To examine the dependence of neural oscillations on GABA_A_ and GABA_B_ inhibitions, we further perform dominant frequency analysis in the ($-v_{sr}^{B},-v_{sr}^{A}$) panel (see Figure 5B). In comparison with the GABA_A_ inhibition, our results suggest that GABA_B_-mediated inhibition plays a dominant role in determining the frequency of neural oscillations generated by the CT system. At a given GABA_A_ inhibition level, increasing the GABA_B_ strength in the TRN-SRN pathway enhances and prolongs the firing depression of SRN neurons. Such strengthened suppression effect leads to a significant reduction in the frequency of neural oscillations. Accordingly, we observe the fast and slow simple neural oscillations at the low and high levels of GABA_B_ inhibition, and the SWDs with frequency varying from 2 to 6.5 Hz at the intermediate level of GABA_B_ inhibition (Figures 5A,B). Consistent with a previous modeling study (Destexhe, 1999), our frequency analysis suggests that the different oscillatory frequencies in the CT system may be mainly attributable to differences in the kinetics of GABA_B_-mediated inhibition in the TRN-SRN pathway.

These results indicate that GABA_A_- and GABA_B_-mediated inhibitions in the TRN-SRN pathway might play combination roles in controlling absence seizures. An intermediate relative strength of GABA_B_ and GABA_A_ inhibitions might trigger the onset of absence seizures in the CT system, and the dominant frequency of SWDs highly depends on the level of GABA_B_ inhibition.

## Discussions

Using a mean-field CT network model, we systematically investigated the roles of thalamic FFI in controlling absence seizures in this study. By computational modeling, we demonstrated that the enhancement of the excitatory Ctx-TRN strength can significantly inhibit typical 2–4 Hz SWDs during absence seizures. Theoretically, this SWD suppression is found to be due to the GABA_B_ weakening effect caused by the collision in TRN neurons. More importantly, we showed that such control manner by the thalamic FFI might be a generalized regulatory mechanism for absence seizures and could be extendable to other pathological factors. Our findings highlight the functional importance of thalamic FFI, which might contribute to the treatment of absence epilepsy.

It has been experimentally reported that loss of the FFI both in thalamus and neocortex might lead to over-excitation of neurons and induce epileptic seizures (Paz and Huguenard, 2015). For example, a recent study has identified a pathophysiological mechanism for the generation of absence seizures in the Gria4^−/−^ mice model of absence epilepsy, for which the TRN receive reduced excitatory inputs from the direct Ctx-TRN neural projections (Paz et al., 2011; Paz and Huguenard, 2015). To a certain extent, this finding is in agreement with our computational observations, but our results established more insightful mechanistic understandings. Similarly, conditionally ablating Ca_v_2.1 channel function in cortical parvalbumin (PV)^+^ interneurons was found to cause a loss of the FFI in neocortex and result in the generalized absence seizures (Rossignol et al., 2013). In addition, a previous experimental study also showed that selective loss of the FFI in rat somatosensory barrel circuits induce epileptic seizures when thalamocortical afferents are activated (Sun et al., 2005). These experimental findings provide clear evidence that the FFI in thalamus and neocortex is of great importance to maintain the balance of excitation and inhibition, which might provide some inspirations to make anti-epileptic drugs (Paz and Huguenard, 2015).

From the anatomical perspective, the GABAergic inhibitions from the TRN to SRN are mediated by both GABA_A_ and GABA_B_ synapses. Several previous experimental studies have suggested that GABAergic transmission within thalamus might play critical roles in shaping abnormal CT oscillations during absence seizures (Liu et al., 1991, 1992; Snead, 1992), but so far the relevant biophysical mechanisms are still not completely established. Our computational findings presented here indicated that GABA_A_ and GABA_B_-mediated inhibitions in the TRN-SRN pathway might play combination roles in regulating absence seizures. For an intermediate relative strength of GABA_B_ and GABA_A_ inhibitions, the double suppression caused by these two types of GABA receptors occurring at different time instants are effective and may provide an effective mechanism to create multiple firing patterns for different neural populations. Mechanistically, such double suppression effect is responsible for the onset of absence seizures in the CT system. Remarkably, our model further makes prediction that the dominant frequency of SWDs in the CT system is more sensitive to GABA_B_-mediated inhibition, and a high level of GABA_B_ inhibition corresponds to a relatively slower oscillation frequency. This observation emphasizes that GABAergic transmission in the thalamic FFI serves as an important intrinsic regulator to modulate the oscillation frequency of SWDs.

The results presented in this work support the idea that significant loss of thalamic FFI might trigger absence seizures (Paz et al., 2011). However, it is worth to notice that the functional roles of the FFI in brain circuits might be diverse and brain region-dependent. In comparison with our current study that enhancing the thalamic FFI could terminate absence seizures, a recent computational study using a basal ganglia-thalamo-cortical network suggested that abnormally strong striatal FFI facilitates to maintain, but not interrupt, synchronous oscillations during absence seizures (Arakaki et al., 2016). Instead, the reduction in the striatal FFI activates medium spiny neurons in the striatum, which might provide potential roles to desynchronize abnormal oscillations and control absence seizures (Arakaki et al., 2016). Moreover, the FFI in neural circuits is not always acting as an opposite depressing the output excitation. Indeed, the FFI might enhance output excitation in some circumstances. For example, as a subset of GABAergic cortical interneurons, the chandelier cells that receive excitatory projections from pyramidal cells. However, it has been observed that these types of interneurons can provide excitatory inputs to their subsequent pyramidal cells (Molnár et al., 2008). Therefore, the functional roles of FFI might highly depend on the involvement of neural circuits and brain regions. Although here we focus on the functional role of thalamic FFI in suppressing absence seizures, it should be noted that the feedback from SRN to TRN might also impact our model dynamics. In additional simulations, we have shown that decreasing the coupling strength of SRN-TRN pathway could push the cortical firing pattern from the SWD oscillation state to the saturation firing state, which is a non-physiological brain state (see Supplementary Figure 1). Further, investigation is needed to uncover the importance of SRN-TRN pathway in controlling absence seizures.

Although our model is designed to capture basic modulations of absence seizures by thalamic FFI, it can be extended to investigate other related issues. For example, deep brain stimulation (DBS) has been demonstrated to be a powerful neurosurgical procedure to treat epileptic seizures (Lega et al., 2010; Fisher and Velasco, 2014; Pantoja-Jiménez et al., 2014). Recent experimental studies have shown that applying high-frequency DBS with suitable current intensity to both SRN and TRN can significantly reduce the occurrence of absence seizures (Lüttjohann and van Luijtelaar, 2013; Pantoja-Jiménez et al., 2014). Nevertheless, so far the most effective thalamic DBS target for treating absence seizures is still debated. In future studies, it is of importance and deserves to use our developed model to determine the most effective thalamic DBS target as well as its corresponding optimal stimulation parameters. On the other hand, several other mental illness, such as the generalized tonic-clonic epilepsy, Parkinson's disease, neurogenic pain and depression, are also reported to be highly associated with the CT dysrhythmia (Llinás et al., 1999). Theoretically, the CT model established in this work might provide a modeling framework for further exploring underlying functional roles of thalamic FFI in these mental illnesses. In addition, considering that suppression of absence seizures in the CT model is a dynamical control process, we will further try to investigate how to control this system from the view of dynamical control in our following work.

To summarize, we have performed mechanistic studies to examine underlying effects of thalamic FFI on the control and modulation of absence seizures. Our results showed that the FFI in thalamus not only contributes to suppressing absence seizures, but also serves as an intrinsic regulator to modulate the oscillation frequency of SWDs generated in the CT system. These observations established an insightful mechanistic understanding on how the thalamic FFI regulates typical 2–4 Hz SWDs during absence seizures, and might provide insightful physiological implications into the treatment of absence seizures.

## Author contributions

MC, DG, YX, and DY conceived and designed the experiments. MC performed the experiments and data analysis. MC and DG wrote the paper.

## Funding

This work was supported by National Natural Science Foundation of China (No. 81571770, No. 81330032, No. 61527815, No. 81371636).

### Conflict of interest statement

The authors declare that the research was conducted in the absence of any commercial or financial relationships that could be construed as a potential conflict of interest.

## Appendix: model equations

The model equations are:

(A1) $$\frac{d\phi_{e}(t)}{dt}=\overset{•}{\phi_{e}}(t)$$

(A2) $$\frac{d\overset{•}{\phi_{e}}(t)}{dt}=\gamma_{e}^{2}[-\phi_{e}(t)+F(V_{e}(t))]-2\gamma_{e}\overset{•}{\phi_{e}}(t)$$

(A3) $$\frac{dV_{e}(t)}{dt}=\overset{•}{V_{e}}(t)$$

(A4) $$\begin{array}{l} \frac{d\overset{•}{V_{e}}(t)}{dt}=\alpha \beta \{-V_{e}(t)+v_{ee}\phi_{e}(t)+v_{ei}F[V_{e}(t)]+v_{es}F[V_{s}(t)]\}\\ \;-\;(\alpha +\beta )\overset{•}{V_{e}}(t) \end{array}$$

(A5) $$\frac{dV_{r}(t)}{dt}\;=\overset{•}{V_{r}}(t)$$

(A6) $$\frac{d\overset{•}{V_{r}}(t)}{dt}=\alpha \beta \{-V_{r}(t)+v_{re}\phi_{e}(t)+v_{rs}F[V_{s}(t)]\}-(\alpha +\beta )\overset{•}{V_{r}}(t)$$

(A7) $$\frac{dV_{s}(t)}{dt}=\overset{•}{V_{s}}(t)$$

(A8) $$\begin{array}{l} \frac{d\overset{•}{V_{s}}(t)}{dt}=\alpha \beta \{-V_{s}(t)+v_{se}\phi_{e}(t)+v_{sr}^{A}F[V_{r}(t)]+v_{sr}^{B}F[V_{r}(t-\tau )]\\ \text{ + }v_{sn}\phi n\text{\}}-(\alpha +\beta )\overset{•}{V_{s}}(t) \end{array}$$

## References

[B1] Arakaki T. Mahon S. Charpier S. Leblois A. Hansel D. (2016). The role of striatal feedforward inhibition in the maintenance of absence seizures. J. Neurosci. 36, 9618–9632. 10.1523/JNEUROSCI.0208-16.2016 27629713 PMC6601939

[B2] Assisi C. Stopfer M. Laurent G. Bazhenov M. (2007). Adaptive regulation of sparseness by feedforward inhibition. Nat. Neurosci. 10, 1176–1184. 10.1038/nn1947 17660812 PMC4061731

[B3] Avoli M. (2012). A brief history on the oscillating roles of thalamus and cortex in absence seizures. Epilepsia 53, 779–789. 10.1111/j.1528-1167.2012.03421.x 22360294 PMC4878899

[B4] Bazhenov M. Timofeev I. Fröhlich F. Sejnowski T. J. (2008). Cellular and network mechanisms of electrographic seizures. Drug Discov. Today Dis. Models 5, 45–57. 10.1016/j.ddmod.2008.07.005 19190736 PMC2633479

[B5] Blumenfeld H. (2005). Cellular and network mechanisms of spike-wave seizures. Epilepsia 46, 21–33. 10.1111/j.1528-1167.2005.00311.x 16302873

[B6] Breakspear M. Roberts J. A. Terry J. R. Rodrigues S. Mahant N. Robinson P. A. (2006). A unifying explanation of primary generalized seizures through nonlinear brain modeling and bifurcation analysis. Cereb. Cortex 16, 1296–1313. 10.1093/cercor/bhj072 16280462

[B7] Chen M. Guo D. Li M. Ma T. Wu S. Ma J.. (2015). Critical roles of the direct GABAergic pallido-cortical pathway in controlling absence seizures. PLoS Comput. Biol. 11:e1004539. 10.1371/journal.pcbi.1004539 26496656 PMC4619822

[B8] Chen M. Guo D. Wang T. Jing W. Xia Y. Xu P.. (2014). Bidirectional control of absence seizures by the basal ganglia: a computational evidence. PLoS Comput. Biol. 10:e1003495. 10.1371/journal.pcbi.1003495 24626189 PMC3952815

[B9] Crunelli V. Leresche N. (2002). Childhood absence epilepsy: genes, channels, neurons and networks. Nat. Rev. Neurosci. 3, 371–382. 10.1038/nrn811 11988776

[B10] Danober L. Deransart C. Depaulis A. Vergnes M. Marescaux C. (1998). Pathophysiological mechanisms of genetic absence epilepsy in the rat. Prog. Neurobiol. 55, 27–57. 10.1016/S0301-0082(97)00091-9 9602499

[B11] Depaulis A. David O. Charpier S. (2016). The genetic absence epilepsy rat from Strasbourg as a model to decipher the neuronal and network mechanisms of generalized idiopathic epilepsies. J. Neurosci. Methods 260, 159–174. 10.1016/j.jneumeth.2015.05.022 26068173

[B12] Depaulis A. Vergnes M. Marescaux C. (1994). Endogenous control of epilepsy: the nigral inhibitory system. Prog. Neurobiol. 42, 33–52. 10.1016/0301-0082(94)90020-5 7480786

[B13] Deransart C. Vercueil L. Marescaux C. Depaulis A. (1998). The role of basal ganglia in the control of generalized absence seizures. Epilepsy Res. 32, 213–223. 10.1016/S0920-1211(98)00053-9 9761322

[B14] Destexhe A. (1998). Spike-and-wave oscillations based on the properties of GABA_B_ receptors. J. Neurosci. 18, 9099–9111. 9787013 10.1523/JNEUROSCI.18-21-09099.1998 PMC6793559

[B15] Destexhe A. (1999). Can GABA_A_ conductances explain the fast oscillation frequency of absence seizures in rodents? Eur. J. Neurosci. 11, 2175–2181. 10.1046/j.1460-9568.1999.00660.x 10336687

[B16] Durón R. M. Medina M. T. Martínez-Juárez I. E. Bailey J. N. Perez-Gosiengfiao K. T. Ramos-Ramírez R.. (2005). Seizures of idiopathic generalized epilepsies. Epilepsia 46, 34–47. 10.1111/j.1528-1167.2005.00312.x 16302874

[B17] Fan D. Wang Q. Perc M. (2015). Disinhibition-induced transitions between absence and tonic-clonic epileptic seizures. Sci. Rep. 5:12618. 10.1038/srep12618 26224066 PMC4519733

[B18] Fisher R. S. Velasco A. L. (2014). Electrical brain stimulation for epilepsy. Nat. Rev. Neurol. 10, 261–270. 10.1038/nrneurol.2014.59 24709892

[B19] Freyer F. Roberts J. A. Becker R. Robinson P. A. Ritter P. Breakspear M. (2011). Biophysical mechanisms of multistability in resting-state cortical rhythms. J. Neurosci. 31, 6353–6361. 10.1523/JNEUROSCI.6693-10.2011 21525275 PMC6622680

[B20] Guo D. Li C. (2009). Stochastic and coherence resonance in feed-forward-loop neuronal network motifs. Phys. Rev. E 79:051921. 10.1103/physreve.79.051921 19518494

[B21] Han H. A. Cortez M. A. Snead O. C. (2012). GABA_B_ receptor and absence epilepsy, in Jasper's Basic Mechanisms of the Epilepsies, 4th Edn., eds Noebels J. Avoli M. Rogawski M. Olsen R. Delgado-Escueta A. (Bethesda: Oxford University Press), 242–256. 10.1093/med/9780199746545.003.0019

[B22] Holt A. B. Netoff T. I. (2013). Computational modeling of epilepsy for an experimental neurologist. Exp. Neurol. 244, 75–86. 10.1016/j.expneurol.2012.05.003 22617489 PMC3769176

[B23] Hu B. Wang Q. (2015). Controlling absence seizures by deep brain stimulus applied on substantia nigra pars reticulata and cortex. Chaos Solitons Fractals 80, 13–23. 10.1016/j.chaos.2015.02.014

[B24] Hu B. Guo D. Wang Q. (2015). Control of absence seizures induced by the pathways connected to SRN in corticothalamic system. Cogn. Neurodyn. 9, 279–289. 10.1007/s11571-014-9321-1 25972977 PMC4427587

[B25] Kee T. Sanda P. Gupta N. Stopfer M. Bazhenov M. (2015). Feed-forward versus feedback inhibition in a basic olfactory circuit. PLoS Comput. Biol. 11:e1004531. 10.1371/journal.pcbi.1004531 26458212 PMC4601731

[B26] Khubieh A. Ratté S. Lankarany M. Prescott S. A. (2016). Regulation of cortical dynamic range by background synaptic noise and feedforward inhibition. Cereb. Cortex 26, 3357–3369. 10.1093/cercor/bhv157 26209846 PMC4961015

[B27] Kros L. Eelkman Rooda O. H. J. De Zeeuw C. I. Hoebeek F. E. (2015a). Controlling cerebellar output to treat refractory epilepsy. Trends Neurosci. 38, 787–799. 10.1016/j.tins.2015.10.002 26602765

[B28] Kros L. Eelkman Rooda O. H. J. Spanke J. K. Alva P. Van Dongen M. N. Karapatis A.. (2015b). Cerebellar output controls generalized spike-and-wave discharge occurrence. Ann. Neurol. 77, 1027–1049. 10.1002/ana.24399 25762286 PMC5008217

[B29] Lega B. C. Halpern C. H. Jaggi J. L. Baltuch G. H. (2010). Deep brain stimulation in the treatment of refractory epilepsy: update on current data and future directions. Neurobiol. Dis. 38, 354–360. 10.1016/j.nbd.2009.07.007 19631750

[B30] Li C. (2008). Functions of neuronal network motifs. Phys. Rev. E 78:037101. 10.1103/physreve.78.037101 18851190

[B31] Lindahl M. Hellgren Kotaleski J. (2016). Untangling basal ganglia network dynamics and function: role of dopamine depletion and inhibition investigated in a spiking network model. eneuro 3:ENEURO.0156-16.2016. 10.1523/ENEURO.0156-16.2016 28101525 PMC5228592

[B32] Liu S. Wang Q. Fan D. (2016). Disinhibition-induced delayed onset of epileptic spike-wave discharges in a five variable model of cortex and thalamus. Front. Comput. Neurosci. 10:28. 10.3389/fncom.2016.00028 27092070 PMC4820438

[B33] Liu Z. Vergnes M. Depaulis A. Marescaux C. (1991). Evidence for a critical role of GABAergic transmission within the thalamus in the genesis and control of absence seizures in the rat. Brain Res. 545, 1–7. 10.1016/0006-8993(91)91262-Y 1650272

[B34] Liu Z. Vergnes M. Depaulis A. Marescaux C. (1992). Involvement of intrathalamic GABA_B_ neurotransmission in the control of absence seizures in the rat. Neuroscience 48, 87–93. 10.1016/0306-4522(92)90340-8 1316571

[B35] Llinás R. R. Ribary U. Jeanmonod D. Kronberg E. Mitra P. P. (1999). Thalamocortical dysrhythmia: a neurological and neuropsychiatric syndrome characterized by magnetoencephalography. Proc. Natl. Acad. Sci. U.S.A. 96, 15222–15227. 10.1073/pnas.96.26.15222 10611366 PMC24801

[B36] Luo C. Li Q. Xia Y. Lei X. Xue K. Yao Z.. (2012). Resting state basal ganglia network in idiopathic generalized epilepsy. Hum. Brain Mapp. 33, 1279–1294. 10.1002/hbm.21286 21520351 PMC6869872

[B37] Lüttjohann A. van Luijtelaar G. (2013). Thalamic stimulation in absence epilepsy. Epilepsy Res. 106, 136–145. 10.1016/j.eplepsyres.2013.03.009 23602552

[B38] Lytton W. W. (2008). Computer modelling of epilepsy. Nat. Rev. Neurosci. 9, 626–637. 10.1038/nrn2416 18594562 PMC2739976

[B39] Marten F. Rodrigues S. Benjamin O. Richardson M. P. Terry J. R. (2009a). Onset of polyspike complexes in a mean-field model of human electroencephalography and its application to absence epilepsy. Philos. Trans. R. Soc. A Math. Phys. Eng. Sci. 367, 1145–1161. 10.1098/rsta.2008.0255 19218156

[B40] Marten F. Rodrigues S. Suffczynski P. Richardson M. P. Terry J. R. (2009b). Derivation and analysis of an ordinary differential equation mean-field model for studying clinically recorded epilepsy dynamics. Phys. Rev. E 79:021911. 10.1103/PhysRevE.79.021911 19391782

[B41] Molnár G. Oláh S. Komlósi G. Füle M. Szabadics J. Varga C.. (2008). Complex events initiated by individual spikes in the human cerebral cortex. PLoS Biol. 6:e222. 10.1371/journal.pbio.0060222 18767905 PMC2528052

[B42] Pantoja-Jiménez C. R. Magdaleno-Madrigal V. M. Almazán-Alvarado S. Fernández-Mas R. (2014). Anti-epileptogenic effect of high-frequency stimulation in the thalamic reticular nucleus on PTZ-induced seizures. Brain Stimul. 7, 587–594. 10.1016/j.brs.2014.03.012 24794164

[B43] Paz J. T. Huguenard J. R. (2015). Microcircuits and their interactions in epilepsy: is the focus out of focus? Nat. Neurosci. 18, 351–359. 10.1038/nn.3950 25710837 PMC4561622

[B44] Paz J. T. Bryant A. S. Peng K. Fenno L. Yizhar O. Frankel W. N.. (2011). A new mode of corticothalamic transmission revealed in the Gria4^−/−^ model of absence epilepsy. Nat. Neurosci. 14, 1167–1173. 10.1038/nn.2896 21857658 PMC3308017

[B45] Paz J. T. Chavez M. Saillet S. Deniau J. M. Charpier S. (2007). Activity of ventral medial thalamic neurons during absence seizures and modulation of cortical paroxysms by the nigrothalamic pathway. J. Neurosci. 27, 929–941. 10.1523/JNEUROSCI.4677-06.2007 17251435 PMC6672924

[B46] Paz J. T. Davidson T. J. Frechette E. S. Delord B. Parada I. Peng K.. (2013). Closed-loop optogenetic control of thalamus as a tool for interrupting seizures after cortical injury. Nat. Neurosci. 16, 64–70. 10.1038/nn.3269 23143518 PMC3700812

[B47] Paz J. T. Deniau J. M. Charpier S. (2005). Rhythmic bursting in the cortico-subthalamo-pallidal network during spontaneous genetically determined spike and wave discharges. J. Neurosci. 25, 2092–2101. 10.1523/JNEUROSCI.4689-04.2005 15728849 PMC6726056

[B48] Pinault D. (2004). The thalamic reticular nucleus: structure, function and concept. Brain Res. Rev. 46, 1–31. 10.1016/j.brainresrev.2004.04.008 15297152

[B49] Potjans T. C. Diesmann M. (2014). The cell-type specific cortical microcircuit: relating structure and activity in a full-scale spiking network model. Cereb. Cortex 24, 785–806. 10.1093/cercor/bhs358 23203991 PMC3920768

[B50] Robinson P. A. Rennie C. J. Rowe D. L. (2002). Dynamics of large-scale brain activity in normal arousal states and epileptic seizures. Phys. Rev. E 65:041924. 10.1103/PhysRevE.65.041924 12005890

[B51] Robinson P. A. Rennie C. J. Rowe D. L. O'connor S. C. (2004). Estimation of multiscale neurophysiologic parameters by electroencephalographic means. Hum. Brain Mapp. 23, 53–72. 10.1002/hbm.20032 15281141 PMC6871818

[B52] Rossignol E. Kruglikov I. Van Den Maagdenberg A. M. J. M. Rudy B. Fishell G. (2013). Ca_V_2.1 ablation in cortical interneurons selectively impairs fast-spiking basket cells and causes generalized seizures. Ann. Neurol. 74, 209–222. 10.1002/ana.23913 23595603 PMC3849346

[B53] Shampine L. F. Thompson S. (2001). Solving DDEs in Matlab. Appl. Num. Math. 37, 441–458. 10.1016/S0168-9274(00)00055-6

[B54] Sloviter R. S. (1991). Feedforward and feedback inhibition of hippocampal principal cell activity evoked by perforant path stimulation: GABA-mediated mechanisms that regulate excitability *in vivo*. Hippocampus 1, 31–40. 10.1002/hipo.450010105 1669342

[B55] Snead O. C. III. (1992). Evidence for GABA_B_-mediated mechanisms in experimental generalized absence seizures. Eur. J. Pharmacol. 213, 343–349. 10.1016/0014-2999(92)90623-C 1319918

[B56] Snead O. C. (1995). Basic mechanisms of generalized absence seizures. Ann. Neurol. 37, 146–157. 10.1002/ana.410370204 7847856

[B57] Sporns O. Kötter R. (2004). Motifs in brain networks. PLoS Biol. 2:e369. 10.1371/journal.pbio.0020369 15510229 PMC524253

[B58] Suffczynski P. Kalitzin S. Lopes Da Silva F. H. (2004). Dynamics of non-convulsive epileptic phenomena modeled by a bistable neuronal network. Neuroscience 126, 467–484. 10.1016/j.neuroscience.2004.03.014 15207365

[B59] Sun Q.-Q. Huguenard J. R. Prince D. A. (2005). Reorganization of barrel circuits leads to thalamically-evoked cortical epileptiform activity. Thalamus Relat. Syst. 3, 261–273. 10.1017/S1472928807000283 18185849 PMC2184932

[B60] Taylor P. N. Thomas J. Sinha N. Dauwels J. Kaiser M. Thesen T.. (2015). Optimal control based seizure abatement using patient derived connectivity. Front. Neurosci. 9:202. 10.3389/fnins.2015.00202 26089775 PMC4453481

[B61] Taylor P. N. Wang Y. Goodfellow M. Dauwels J. Moeller F. Stephani U.. (2014). A computational study of stimulus driven epileptic seizure abatement. PLoS ONE 9:e114316. 10.1371/journal.pone.0114316 25531883 PMC4273970

[B62] Tepper J. M. Wilson C. J. Koós T. (2008). Feedforward and feedback inhibition in neostriatal GABAergic spiny neurons. Brain Res. Rev. 58, 272–281. 10.1016/j.brainresrev.2007.10.008 18054796 PMC2562631

[B63] Tolaymat A. Nayak A. Geyer J. D. Geyer S. K. Carney P. R. (2015). Diagnosis and management of childhood epilepsy. Curr. Probl. Pediatr. Adolesc. Health Care 45, 3–17. 10.1016/j.cppeds.2014.12.002 25720540

[B64] van Albada S. J. Robinson P. A. (2009). Mean-field modeling of the basal ganglia-thalamocortical system. I: firing rates in healthy and parkinsonian states. J. Theor. Biol. 257, 642–663. 10.1016/j.jtbi.2008.12.018 19168074

[B65] van Albada S. J. Gray R. T. Drysdale P. M. Robinson P. A. (2009). Mean-field modeling of the basal ganglia-thalamocortical system. II: dynamics of parkinsonian oscillations. J. Theor. Biol. 257, 664–688. 10.1016/j.jtbi.2008.12.013 19154745

[B66] Womelsdorf T. Valiante T. A. Sahin N. T. Miller K. J. Tiesinga P. (2014). Dynamic circuit motifs underlying rhythmic gain control, gating and integration. Nat. Neurosci. 17, 1031–1039. 10.1038/nn.3764 25065440

[B67] Yang D.-P. Mckenzie-Sell L. Karanjai A. Robinson P. A. (2016). Wake-sleep transition as a noisy bifurcation. Phys. Rev. E 94:022412. 10.1103/PhysRevE.94.022412 27627340

[B68] Yuste R. (2015). From the neuron doctrine to neural networks. Nat. Rev. Neurosci. 16, 487–497. 10.1038/nrn3962 26152865

