# Supplementary material for: Control of Absence Seizures by the Thalamic Feed-Forward Inhibition
Source: Front Comput Neurosci. 2017 Apr 26;11:31. doi: 10.3389/fncom.2017.00031 (PMC5405150; doi:10.3389/fncom.2017.00031)
Supplement: Supplementary file 1 [file Image1.PDF]

Supplementary Material

Control of Absence Seizures by the Thalamic Feed-forward Inhibition

Mingming Chen [1,†], Daqing Guo[1,2,*,†], Yang Xia[1,2], Dezhong Yao[1,2,*]

*** Correspondence:** Daqing Guo (dqguo@uestc.edu.cn)

Dezhong Yao (dyao@uestc.edu.cn)

Supplementary Figures

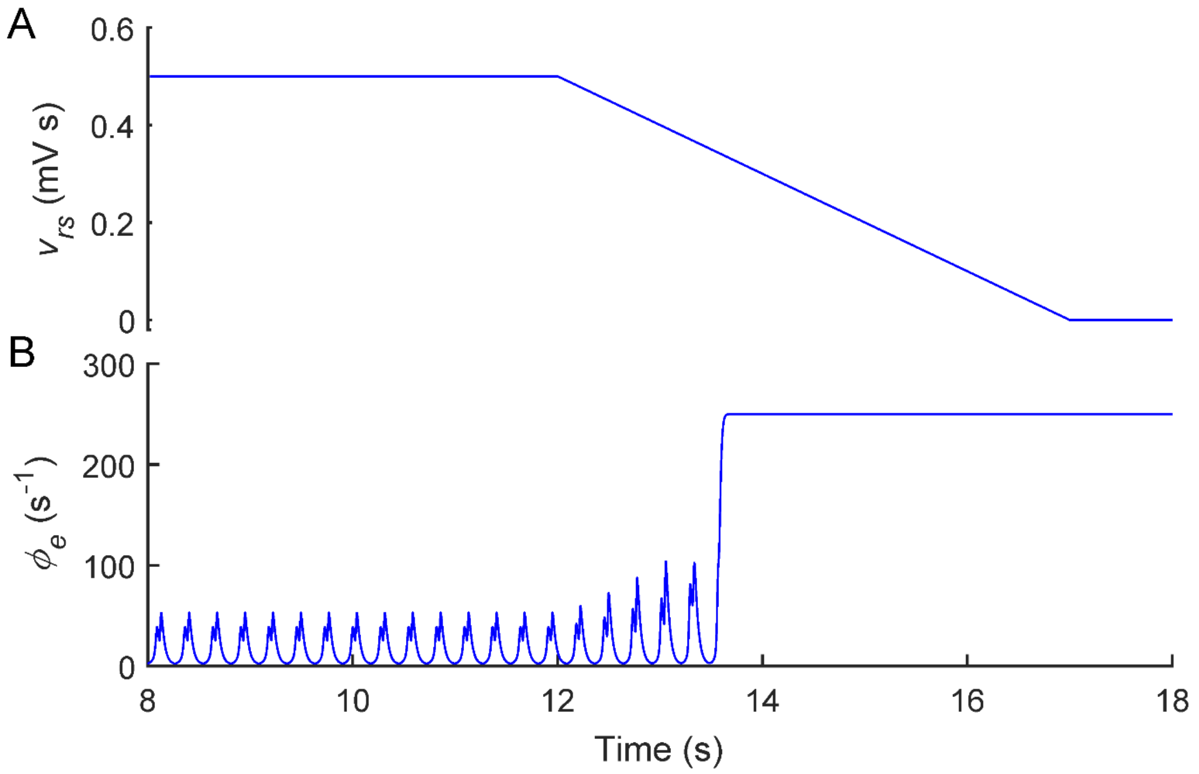

Supplementary Figure. 1. The effects of decreasing the coupling strength v_{rs} on controlling absence seizures. (A) Linearly decreasing the coupling strength v_{rs}. (B) The cortical firing patterns change from SWD oscillation state to saturation firing state with v_{rs} decreasing to 0.
